# Ultrasound in children and adolescents with inflammatory bowel disease

**DOI:** 10.1186/1824-7288-40-S1-A28

**Published:** 2014-08-11

**Authors:** Francesco La Seta, Michele Citrano, Lorenzo Tesè, Lucrezia Bruno

**Affiliations:** 1 Dept. of Radiology, ‘V. Cervello’ Hospital - A.O.R. ‘Villa Sofia – Cervello, Palermo, Italy; 2 Dept of Pediatrics, ‘V. Cervello’ Hospital - A.O.R. ‘Villa Sofia – Cervello, Palermo, Italy; 3 Specialty in Pediatrics, University of Palermo, Italy

## 

The term *Inflammatory Bowel Disease (IBD)* is used to describe several idiopathic gastrointestinal disorders; among these diseases, the most commonly encountered in clinical practice are Crohn’s Disease (CD) and Ulcerative Colitis (UC).

CD and UC are different both in distribution of gastrointestinal tract involvement and depth of inflammation. 25% of patients reveals IBD during childhood or adolescence. [1]

No consensus exists regarding the optimal techniques and imaging modalities when evaluating IBD, especially in the paediatric age. [2] The correct imaging choice often depends on clinical presentation of patients. The most common situations are: the initial diagnosis of suspected IBD; patients with known IBD presenting with acute symptoms; follow-up of patients with or without new symptoms. [2]

Multiple methods can be used to diagnose and examine paediatric patients with IBD, including Computed Tomography (CT), Magnetic Resonance (MR), Small Bowel Follow-through radiographic examination (SBFT), Endoscopy, and Ultrasound (US). [3,4]

While UC is mainly clinically managed with endoscopy and US, CD very often needs cross-sectional imaging to assess the exact distribution and activity of the disease, and to detect extraluminal complications (phlegmon, abscess, fistula, bowel perforation).

Due to early onset of disease, paediatric patients obviously need additional attention to minimize radiation exposure: it is mandatory to avoid unnecessary ionizing radiation techniques in IBD imaging. [2-4]

Consequence of the foregoing statements, US and (more recently) MR-enterography have become the preferred imaging modalities in management of young patients with IBD. [2-6]

In clinical practice, in Europe, US is currently the first examination requested to evaluate patients with abdominal symptoms. US is accurate in diagnosis of several paediatric (intestinal and non-intestinal) diseases; US may also help when selecting patients needing additional caring among children suffering from gastroenteric tract diseases. [5] Furthermore, US may offer information that are difficult to obtain with other diagnostic modalities, and in fact it is the only technique which could possibly investigate in real time all the followings: the morphological and functional aspects of the bowel wall, the vascularisation (with color-Doppler) of thickened bowel, the stiffness of intestinal loops, and perivisceral spaces. [5]

The aim of this review is to elucidate the current role of US in diagnosis and follow-up of paediatric patients with UC and CD, and to discuss the advantages and disadvantages of this radiation-free imaging methods in terms of cost, safe, diagnostic accuracy and accessibility compared with other diagnostic modalities.
